# Chemotherapy-based gonadotoxicity risk evaluation as a predictor of reproductive outcomes in post-pubertal patients following ovarian tissue cryopreservation

**DOI:** 10.1186/s12905-021-01343-z

**Published:** 2021-05-13

**Authors:** Gilad Karavani, Amihai Rottenstreich, Natali Schachter-Safrai, Adiel Cohen, Michael Weintraub, Tal Imbar, Ariel Revel

**Affiliations:** 1 Department of Obstetrics and Gynecology, Hadassah Ein-Kerem Medical Center and Faculty of Medicine, Hebrew University of Jerusalem, Jerusalem, Israel; 2 Faculty of Medicine, Hebrew University of Jerusalem, Jerusalem, Israel; 3 Department of Pediatric Hematology - Oncology, Hadassah Hebrew University Hospital, Jerusalem, Israel; 4 Infertility and IVF Unit, Department of Obstetrics and Gynecology, Hadassah Ein-Kerem Medical Center and Faculty of Medicine, Hebrew University of Jerusalem,, Jerusalem, Israel

**Keywords:** Ovarian tissue cryopreservation, Fertility preservation, Chemotherapy, Ovarian auto-transplantation, Cancer survivors

## Abstract

**Background:**

The sterilizing effect of cancer treatment depends mostly on the chemotherapy regimen and extent of radiotherapy. Prediction of long-term reproductive outcomes among cancer survivors according to chemo-radiotherapy regimen may improve pre-treatment fertility preservation counseling and future reproductive outcomes.

**Methods:**

The aim of this study was to evaluate long term reproductive outcomes in cancer survivors according to gonadotoxicity risk estimation of the chemo-radiotherapy regimens utilized. This retrospective cohort study was comprised of post-pubertal female patients referred for fertility preservation during 1997 and 2017 was performed. Eligible adult patients were addressed and asked to complete a clinical survey regarding their ovarian function, menstruation, reproductive experience and ovarian tissue auto-transplantation procedures. Results were stratified according to the gonadotoxic potential of chemotherapy and radiotherapy they received—low, moderate and high-risk, defined by the regimen used, the cumulative dose of chemotherapy administered and radiation therapy extent.

**Results:**

A total of 120 patients were eligible for the survey. Of those, 92 patients agreed to answer the questionnaire. Data regarding chemotherapy regimen were available for 77 of the 92 patients who answered the questionnaire. Menopause symptoms were much more prevalent in patients undergoing high vs moderate and low-risk chemotherapy protocol. (51.4% vs. 27.3% and 16.7%, respectively; *p* < 0.05). Spontaneous pregnancy rates were also significantly lower in the high-risk compared with the low-risk gonadotoxicity regimen group (32.0% vs. 58.3% and 87.5%, respectively; *p* < 0.05).

**Conclusion:**

Patients scheduled for aggressive cancer treatment have significantly higher rates of menopause symptoms and more than double the risk of struggling to conceive spontaneously. Improving prediction of future reproductive outcomes according to treatment protocol and counseling in early stages of cancer diagnosis and treatment may contribute to a tailored fertility related consultation among cancer survivors.

## Background

The long-term survival rate of cancer patients increases, coupled with the demand for fertility preservation to enable parenthood. The sterilizing effect of chemotherapy depends mostly upon whether it consists of alkylating agents [1–3] and other high-risk chemotherapy, and extent of radiotherapy, in addition to the patient's age at chemotherapy initiation. There is an increasing number of publications aiming to evaluate and quantify the gonadotoxicity profile of different chemotherapy regimens in order to predict the effect on ovarian function, future fertility and early iatrogenic menopause [4–9].

Abdominal-pelvic radiation therapy has an additional negative effect, dependent on the extent of the radiation field and radiation cumulative dose, on fertility via direct damage to the ovaries [10], which are more vulnerable as compared to other tissues. High dose cranial irradiation damaging the hypothalamic-pituitary axis may result in hypogonadotropic hypogonadism [11], though mostly temporary. The cumulative dose administered is an additional risk factor [12].

Primary ovarian insufficiency (POI) is defined by cessation of ovarian function prior to the age of 40, with or without ovarian follicle depletion. It is characterized by the presence of oligomenorrhea or amenorrhea, elevated gonadotropins and low estradiol levels. The overall risk for ovarian insufficiency in patients who underwent gonadotoxic treatment, was shown to be 30% after a mean follow up of 50 months [13]. Guidelines advocate counselling young cancer patients on the possible gonadotoxic effect of chemotherapy and the options available for fertility preservation [12]. The suggested options for fertility preservation in women include cryopreservation of oocyte, embryos and ovarian tissue [14].

The use of gonadotropin-releasing hormone agonist (GnRHa) during chemotherapy has been shown to reduce chemotherapy induced ovarian insufficiency. Indeed, recent data [15] and the updated ASCO guidelines support its use when proven fertility preservation methods are not feasible and particularly in breast cancer patients, and this strategy is now recommended as an available option in this setting [16, 17]. An additional method for fertility preservation in women prior to pelvic radiotherapy is surgical ovarian transposition and this method may be offered in attempt to prevent POI [18].

Ovarian tissue cryopreservation (OTCP) has been introduced over two decades ago [19]. In this procedure, the ovary is partially resected (partial oophorectomy) or removed completely laparoscopically and immediately processed in the laboratory, where the ovarian cortex is carefully separated from the medulla and cut into cortex stripes which are transferred to a freezing medium for cryopreservation and future thawing for auto-transplantation. The growing experience along with documented success rates have established the OTCP procedure as a valid fertility preservation option in some countries while it is still considered experimental in others [14].

Two of the most prominent advantages of OTCP are that it is the main option for pre-pubertal girls which are not suitable for oocytes retrieval and the ability to perform the procedure almost immediately, without preceding hormonal treatment, thus obviating the need to postpone chemotherapy initiation. However, this procedure requires an abdominal surgery with possible surgical complications, affects the ovarian reserve and enables mainly the preservation of primordial and primary follicles that will survive the cryopreservation and thawing.

The utility of OTCP was evaluated according to the type of malignancy in few studies [13, 20] with additional assessment of post treatment ovarian function that demonstrated better results in patients diagnosed with breast cancer.

The objective of our study was to compare the long-term reproductive outcomes in a large cohort of patients who underwent OTCP according to the expected gonadotoxic risk. This information should improve counseling and expectation management for cancer survivors. This is of particular importance as current literature regarding gonadotoxicity is relatively heterogenous in terms of the fertility preservation procedures evaluated, with limited data regarding OTCP.

## Methods

### Setting

At Hadassah university affiliated hospital, we have been providing fertility preservation services for more than two decades. Patients diagnosed with cancer in their reproductive years are referred to our clinic for counselling regarding fertility preservation options. Referral rates have increased over the two decades since fertility preservation was introduced. Oncologists in our institution discuss fertility preservation with their adult patients before initiating cancer treatment and refer those who wish to freeze ovarian tissue or oocytes to our unit.

### Participants

A retrospective review of a prospectively-collected database. The study population included post-pubertal female patients diagnosed with cancer who underwent OTCP at Hadassah In-Vitro Fertilization (IVF) unit, during 1997–2017 with at least a 12-month interval from completion of chemotherapy or radiotherapy. Survivors, including those who still had material stored and those who had used or discarded their material, were eligible to participate and were contacted by telephone. Deceased patients, patients currently under the age of 18 and patients with lack of contact data were excluded from the study.

### Data source and long-term follow-up

A study-specific questionnaire was developed by the researchers. The questionnaire included questions with fixed response options about baseline characteristics (age at cancer diagnosis and currently, relationship status at cancer diagnosis and currently); the type of cancer; year of storage; whether a doctor had been consulted about fertility since storing material (yes/no) and outcome of consultation (was told still fertile/not fertile but took no further steps/not fertile and sought help with conception/other); menstruation status and whether menstrual period patterns had changed since undergoing cancer treatment; whether the stored material had been used (yes/no); reasons for not using the stored material (multiple response options from a list); outcomes of using stored material (thawing unsuccessful, thawing successful but no embryos, embryos formed but no pregnancy, pregnancy loss, currently pregnant/live birth); reproductive experiences before and after storing material and whether material was still stored.

The electronic medical record database of the Reproductive Medicine and IVF unit in our medical center was reviewed between January and March 2018. We reviewed the records of all patients who underwent OTCP at our university-affiliated medical center during 1997–2017. The following data were extracted: demographics, age at preservation, age of menarche, indication for OTCP, type of cancer and chemotherapy treatment prior to OTCP. Women who passed away (n = 49) during follow-up were excluded from the study. Information regarding deceased patients was acquired through patient’s file and this information was later confirmed by the national death registry.

Data regarding malignancy type was additionally collected and defined as previously reported [21, 22]: (1) Lymphoma, (2) Leukemia, (3) benign hematologic conditions in need of bone marrow transplantation (mostly thalassemia and immunodeficiency conditions), (4) sarcoma, (5) breast cancer, (6) other solid tumors (carcinoma) and (7) other malignancies including blastomas, central nervous system and neuroendocrine malignancies.

Ovarian function was assessed by the questionnaire using questions regarding time interval from chemotherapy and the resumption and persistence of regular menstrual cycles. Additional data collected included menopause symptoms assessment (graded 1–5): Hot flushes, night sweats, vaginal dryness, changes in cycle duration and bleeding, low mood and irritability. A patient with a mean symptoms grade of more than 2 was considered as suffering from menopause symptoms.

Irregular menses was defined as more than 15 days (in average) difference between menses. Absence of menstruation for at least 3 consecutive cycles (3 months in a woman who previously had regular menstruations) or for at least 6 consecutive months (if she had irregular menstruations) was defined as amenorrhea.

Ovarian function and reproductive outcomes in patients that underwent ovarian tissue auto-transplantation were assessed and analyzed according to their condition prior to the auto-transplantation.

Patients were contacted by telephone. The questionnaire was presented and filled after consent by patients over the age of 18 with at least 12 months interval from completion of chemotherapy or radiotherapy. All questionnaires were conducted by a single dedicated physician (A.C). Institutional review board approval, was obtained for this study (IRB 0288–16-HMO, approved in July 2016). Each patient gave an oral informed consent prior to answering the questionnaire.

### Allocation to gonadotoxicity risk groups by treatment protocol

For analysis and future consultation purposes, we categorized our patients into 3 risk groups—low, moderate and high. Low, moderate and high-risk categories were defined according to the estimated risk for gonadotoxicity based on the overall chemotherapy agents received as well as the field and intensity of radiation therapy as previously established [8, 9, 23].

Risk assignment was based on the following factors:

(1) The type of chemotherapy used, specifically the inclusion of alkylating agents and other high-risk gonadotoxic agents in the regimen (Cyclophosphamide, Ifosphamide, Nitrogen mustard, Procarbazine, Chlorambucil etc. [8], (2) The cumulative dose of alkylating agents and the alkylator equivalent score [9] and (3) Radiation therapy to the pelvis, abdomen, cranium or total body irradiation [23].

Patients were considered as having high-risk for gonadotoxicity in case they received one of the abovementioned or similar high-risk gonadotoxic agents in an overall cumulative dose known to potentially affect the gonads, as previously described by Green et al. [9]. Additionally, patients who received radiotherapy treatments that are associated with a high-risk of amenorrhea were also considered as high-risk, as followed: Post-pubertal girls receiving pelvic or whole abdominal radiation dose > 10 Gy, adult women receiving pelvic or whole abdominal radiation dose > 6 Gy or total Body Irradiation (prior to stem cell or bone marrow transplantation), as previously described by Green et al. [9]. Patients that underwent aggressive chemo-radiotherapy due to the need of bone marrow transplantation were also defined as high-risk. Craniospinal irradiation alone was not considered as high gonadotoxicity risk.

Patients were considered as having a moderate-risk for gonadotoxicity in case they received Cispaltin, Carboplatin, Adriamycin or a low cumulative dose of one of the abovementioned or similar high-risk gonadotoxic agents as previously described by Green et al. [9]. Post-pubertal girls receiving pelvic radiation or whole abdominal dose of 5–10 Gy or craniospinal irradiation dose of > 25 Gy [23] without additional treatment with a high-risk cytotoxic agent were also defined as having moderate gonadotoxicity risk.

Low-risk gonadotoxicity group included patients who received Bleomycin, Actinomycin D, Vincristine, Methotrexate or 5-Fluorouracil and similar agents considered as having low gonadotoxic risk [8, 9], and those who received low dose radiation in areas other than craniospinal, pelvic or abdomen fields.

### Preserved ovarian tissue usage index

Patients with previous or present desire and attempt to conceive who responded to our questionnaire were defined as positive *"Attempted pregnancy".*

*"Tissue usage index"* was calculated as the rate of patients that underwent at least one auto-transplantation out of the group of patients with known follow-up (either passed away or pubertal cancer survivors).

### Statistical analysis

Patient characteristics are described as proportions for categorical variables and medians and interquartile range for continuous variables without a normal distribution. Continuous data is presented as mean ± standard deviation (SD) and calculated by the student’s *t* test. Significance between groups was assessed by the Chi square test and Fisher's exact test for categorical variables and the Mann–Whitney U test for continuous variables. SPSS (for Windows software, version 19; IBM Corp) was used for statistical analysis. A 2-sided *P* value < 0.05 indicated statistical significance.

## Results

Two hundred thirty-one patients underwent OTCP during 1997 and 2017 in our medical center. After exclusion of 21 pre-pubertal girls, 49 deceased patients, 18 patients that were lost to follow-up and 23 patients with current active disease, 120 adult patients (52.0%) were suitable for the survey. Out of those, 11 patients (9.2%) refused to answer the questionnaire and 17 patients (14.2%) were not available by telephone after several phone-call attempts. Overall, 92 out of 120 patients agreed to participate in the study and answered our survey—achieving a 76.7% response rate. Data regarding chemotherapy and radiotherapy were available for 77 out of 92 patients with long term follow-up and these patients were included in the final analysis.

The mean age at OTCP was 23.7 ± 6.3 years (range 13–40 years). The mean duration of follow up was 9.4 ± 5.4 years, with a mean age of 33.2 ± 8.4 years (range: 18–48 years) at the end of follow-up period. All patients included were post-pubertal at the time of diagnosis. Indications for OTCP were planned chemotherapy or radiotherapy due to malignant conditions or planned bone marrow transplantation with possible induced ovarian toxicity. Malignancies included: lymphoma, leukemia, benign hematologic conditions such as thalassemia and immune-deficiency, breast cancer, sarcoma and carcinoma or other solid malignancies. Additional baseline characteristics of the study population are presented in Table 1.

**Table 1:** Basic characteristics of cancer survivors who responded and filled the follow-up questionnaire (n = 77)

Parameter	
Age at OTCP (years)	23.7 ± 6.3
Type of malignancy	
Lymphoma	37/77 (48.1%)
Leukemia	9/77 (11.7%)
Benign hematologic	4/77 (5.2%)
Breast cancer	8/77 (10.4%)
Sarcoma	10/77 (13.0%)
Carcinoma	5/77 (6.5%)
Other^a^	4/77 (5.2%)
GnRHa during chemotherapy	10/77 (13.0%)
Chemotherapy prior to OTCP	21/77 (27.3%)
Risk for chemotherapy induced gonadotoxicity^b^	
Low risk	18/77 (23.4%)
Moderate risk	22/77 (28.6%)
High risk	37/77 (48.1%)

Data are given as n(%) or mean ± SD

*OTCP*, ovarian tissue cryopreservation; *GnRHa*, Gonadotropin releasing hormone agonist

^a^Other tumors included blastomas, central nervous system and neuro-endocrine tumors

^b^Gonadotoxicity risk was determined by the following factors: (1) Type of chemotherapy used, specifically the inclusion of alkylating agents in the regimen, (2) The cumulative dose of alkylating agents and the alkylator equivalent score and (3) Radiation therapy to the pelvis, abdomen, cranium or total body irradiation

### Ovarian function and reproductive outcomes

Out of 77 patients, the rate of irregular menstruation or oligomenorrhea was 39.0% (30/77 patients). The rate of patients experiencing menopause symptoms was significantly higher in the high-risk compared to the moderate and low-risk groups (51.4% vs 27.3% and 16.7%, respectively; p < 0.05). A similar trend, though not significant, was noted in both amenorrhea (24.3% vs 18.2% and 16.7%, respectively; NS) and menstrual irregularity rates (51.4% vs 31.8% and 22.2%, respectively; NS).

No significant differences were found in age at treatment, chemotherapy prior to OTCP, GnRHa treatment during chemotherapy or follow-up time (time lapsed from treatment to questionnaire) between the low, moderate and high-risk groups (Data presented in Table 2).

**Table 2:** Fertility outcomes and ovarian tissue usage according to chemotherapy induced gonadotoxicity risk (n = 77)

Parameter	Risk for chemotherapy induced gonadotoxicity^a^		
	Low risk	Moderate risk	High risk
N	18	22	37
Age at treatment	20.1 + 4.2 (19)	22.5 + 5.7 (22)	26.1 + 6.5 (24)
Chemotherapy prior to OTCP	4/18 (22.2%)	4/22 (18.2%)	13/38 (34.2%)
GnRHa during treatment	0/18 (0%)	2/22 (9.1%)	6/37 (16.2%)
Follow-up time (years)	9.0 + 6.2 (8)	9.8 + 4.5 (10)	9.6 + 5.6 (8)
Menopause symptoms^b^	3/18 (16.7%)	6/22(27.3%)	19/37(51.4%)
Menstrual irregularity	4/18 (22.2%)	7/22 (31.8%)	19/37 (51.4%)
Amenorrhea	3/18 (16.7%)	4/22 (18.2%)	9/37 (24.3%)
Attempt to conceive	8/18 (44.4%)	12/22 (54.6%)	25/37 (67.6%)
Spontaneous pregnancy^b^	7/8 (87.5%)	7/12 (58.3%)	8/25 (32.0%)
IVF treatment (n of patients)	0	5	15
IVF pregnancy	N/A	2/5 (40.0%)	4/15 (26.7%)
Tissue usage index^c^	N/A	16.7% (2/12)	20.0% (5/25)
Ovarian tissue auto-transplantation pregnancy	N/A	1/2 (50.0%)	3/5 (60.0%)
Oocyte donation pregnancy	N/A	2/5 (40.0%)	2/15 (13.3%)

Data are given as n(%), n/N(%) or mean ± SD (median)

*OTCP,* ovarian tissue cryopreservation; *IVF,* In-vitro fertilization

^a^Risk assignment was based on the following factors: (1) Type of chemotherapy used, specifically the inclusion of alkylating agents in the regimen, (2) The cumulative dose of alkylating agents and the alkylator equivalent score and (3) Radiation therapy to the pelvis, abdomen, cranium or total body irradiation

^b^*P* value < 0.05

^c^Calculated by the number of patients that underwent auto-transplantation divided by number of patients desiring pregnancy

Out of 45 patients that attempted to conceive, 22 patients (48.9%) achieved spontaneous pregnancies, 6 patients (13.3%) became pregnant through IVF treatment (using autologous fresh or frozen oocytes) and 14 patients failed to conceive even after several IVF attempts (the remaining 3 patients are either currently trying to spontaneously conceive or in the process of fertility counseling towards fertility treatment). Seven out of these 14 patients have undergone an ovarian tissue auto-transplantation achieving 4 clinical pregnancies leading to 4 live births. Two patients from the moderate risk group and two patients from the high-risk group who failed to conceive through IVF using autologous oocytes or auto-transplantation attempted and conceived through oocyte donation cycles (1–3 cycles).

Spontaneous pregnancy rates were significantly reduced in patients that received high vs. moderate and low-risk chemotherapy protocol (32.0% vs. 58.3% and 87.5%, respectively; p < 0.05). The rates of IVF pregnancies, tissue usage index and pregnancies following ovarian tissue auto-transplantation did not significantly differ between the groups (Table 2).

## Discussion

This study aimed to address the possible prediction of long-term reproductive outcomes in cancer survivors in relation to their treatment protocol. Our findings show significantly lower rates of spontaneous pregnancies and an increased need of assisted reproductive technology (ART) in order to conceive in the high-risk group. The IVF success rates in the high-risk group were additionally low. Moreover, patients that received treatment protocol defined as having a high risk for gonadotoxicity were more likely to experience menopause symptoms compared with those receiving moderate and low-risk treatment protocols.

Our data are based on a long term follow up of a large group of cancer survivors with additional data collected by questionnaires. Risk-based assessment according to the chemotherapeutic agent and radiation used, was found as a significant predictor of patient reproductive outcomes.

The non-renewable primordial follicles constituting the ovarian reserve are particularly vulnerable to cytotoxic exposure. Certain chemotherapeutic agents, such as alkylating agents have been recognized as having more detrimental impact on ovarian follicles [8, 9, 24]. In addition, the primordial follicles are very sensitive to radiation exposure in a dose-dependent manner [8, 24].

Poor results of ovarian stimulation in IVF cycles following chemotherapy were previously described by Dolmans et al. [25]. Accordingly, the marked loss in the number of follicles caused by the cytotoxic effect on primordial follicles during chemotherapy may explain the unfavorable results of ovarian stimulation [21] shown in the gonadotoxicity high-risk group in our study.

Therefore, in this study we consulted oncology and pediatric hematology- oncology specialists and created a risk-based evaluation of cancer patients based on the chemotherapy regimen and radiation extent and dose used according to previous studies [8, 9, 23] We have shown a significant association between treatments with higher risk of gonadotoxicity and consequent lower ovarian function, spontaneous pregnancy and successful IVF pregnancy rates.

This risk evaluation is easily designated, facilitating its potential use for improved pre-treatment evaluation, counseling regarding ovarian cryopreservation and future reproductive outcomes prediction.

Women need to be aware that fertility preservation may not be an insurance against infertility but rather that it might add to the chance of achieving parenthood in the future. Women should also be informed that, although natural conception may be possible, they are at risk of premature menopause as a consequence of the cancer treatment.

As the number of cancer patients undergoing OTCP is expected to continue to grow, better prediction of future reproductive outcomes is essential. Our study provides patients and their families with data regarding the potential fertility threat associated with cancer therapy and cryopreserved tissue usage index in regard to malignancy type and planned chemotherapy and radiotherapy treatment regimen. It is worth noting that we aimed to focus on outcomes of women undergoing OTCP, as including those who underwent other procedures for fertility preservation may a potential bias. In this regard, the lack of differences in women's characteristics between the low, moderate and high-risk groups, may stem from the inclusion of only those undergoing OTCP. This difference compared to previous publications, may be at least partially accounted for by the inclusion of various fertility preservation altogether in the current literature.

Finally, as the current cohort included only female patients, further studies focusing on male gonadotoxicity are warranted.

It was previously reported that while eight percent of cancer patients may be interested in fertility preservation, some oncologists never refer these patients to a reproductive specialist and that female cancer patients feel poorly informed in this regard [26]. This further highlights the paramount importance of multidisciplinary management and close collaboration between the oncology and reproductive professionals in the management of these patients. It is worth noting that OTCP is not covered by the medical insurance in most countries and is associated with a substantial financial burden for patients, thus limiting its widespread utilization.

The retrospective study design raises several biases inherent to such data collection. the collection of some of the data by self-reporting is a potential caveat that raises the possibility of interviewer and response biases and recall bias. An additional limitation is a possible selection bias, as our cohort included only OTCP cases without a control group of patients the underwent other fertility preservation methods. Sonographic and laboratory evaluation of ovarian function were lacking and only 10 patients received GnRHa treatment during chemotherapy, a number too small to accurately identify a difference in this parameter Furthermore, the relatively small sample size and the lack of validated measures of patient reported outcomes are additional caveats as well as the definition of amenorrhea using relatively short time periods (3–6 months), with a possible inclusion of transient amenorrhea cases. Nevertheless, the major strength of the current investigation is its relatively long follow-up period. Finally, while acknowledging the aforementioned caveats, we believe that the study findings serve as an important contribution to current knowledge as it particularly evaluated the outcomes of OTCP as compared to current literature which is composed of a relatively heterogenous population of women in terms of the fertility preservation procedures included.

## Conclusions

In conclusion, this study demonstrates that risk assessment of cancer survivors based on the chemotherapy and radiation used, could serve in order to predict the reproductive outcomes and the possible need for ART. This study suggests that a substantial portion of patients that receive gonadotoxic high-risk regimen will suffer from menopause symptoms and will require further infertility treatments. These findings may aid in the pre-treatment counseling as well as the follow-up care and contribute to improved fertility outcomes.

## Data Availability

The datasets used and/or analyzed during the current study are available from the corresponding author on reasonable request.
